# Abnormal Brain Development in Huntington’ Disease Is Recapitulated in the zQ175 Knock-In Mouse Model

**DOI:** 10.1093/texcom/tgaa044

**Published:** 2020-08-05

**Authors:** Chuangchuang Zhang, Qian Wu, Hongshuai Liu, Liam Cheng, Zhipeng Hou, Susumu Mori, Jun Hua, Christopher A Ross, Jiangyang Zhang, Peggy C Nopoulos, Wenzhen Duan

**Affiliations:** 1 Division of Neurobiology, Department of Psychiatry and Behavioral Sciences, Johns Hopkins University School of Medicine, Baltimore, MD 21287, USA; 2 Department of Medicine, Beijing University of Chinese Medicine, Beijing 100029, China; 3 Department of Radiology, Johns Hopkins University School of Medicine, Baltimore, MD 21205, USA; 4 F.M. Kirby Research Center for Functional Brain Imaging, Kennedy Krieger Institute, Baltimore, MD 21205, USA; 5 Department of Neuroscience, Johns Hopkins University School of Medicine, Baltimore, MD 21285, USA; 6 Department of Pharmacology and Molecular Sciences, Johns Hopkins University School of Medicine, Baltimore, MD 21205, USA; 7 Department of Neurology, Johns Hopkins University School of Medicine, Baltimore, MD 21205, USA; 8 Deaprtment of Radiology, New York University Grossman School of Medicine, New York City, NY 10016, USA; 9 Departments of Psychiatry, Neurology, Pediatrics, University of Iowa Carver College of Medicine, Iowa city, IA 52242, USA; 10 Program in Cellular and Molecular Medicine, Johns Hopkins University School of Medicine, Baltimore, MD 21205, USA

**Keywords:** abnormal brain development, brain atrophy, Huntington’s disease, mouse model, MRI

## Abstract

Emerging cellular and molecular studies are providing compelling evidence that altered brain development contributes to the pathogenesis of Huntington’s disease (HD). There has been lacking longitudinal system-level data obtained from *in vivo* HD models supporting this hypothesis. Our human MRI study in children and adolescents with HD indicates that striatal development differs between the HD and control groups, with initial hypertrophy and more rapid volume decline in HD group. In this study, we aimed to determine whether brain development recapitulates the human HD during the postnatal period. Longitudinal structural MRI scans were conducted in the heterozygous zQ175 HD mice and their littermate controls. We found that male zQ175 HD mice recapitulated the region-specific abnormal volume development in the striatum and globus pallidus, with early hypertrophy and then rapidly decline in the regional volume. In contrast, female zQ175 HD mice did not show significant difference in brain volume development with their littermate controls. This is the first longitudinal study of brain volume development at the system level in HD mice. Our results suggest that altered brain development may contribute to the HD pathogenesis. The potential effect of gene therapies targeting on neurodevelopmental event is worth to consider for HD therapeutic intervention.

## Introduction

Huntington’s disease (HD) is caused by a CAG trinucleotide repeat expansion in the huntingtin gene (*HTT*), which encodes an expanded polyglutamine stretch in the huntingtin protein (HTT). Cumulative cellular and molecular studies have demonstrated that brain developmental abnormalities may be a substrate for impaired brain function and later neurodegeneration in HD (Mehler and Gokhan 2000; Reiner et al. 2003; Cattaneo et al. 2005; Godin et al. 2010; Molina-Calavita et al. 2014; Conforti et al. 2018; Wiatr et al. 2018; Barnat et al. 2020). The HD brain pathology may therefore represent, at least partially, the consequences of abnormal development. Although HTT is widely expressed ubiquitously, the mutation only causes the demise of selective neuronal subtypes. The previous study indicated that mutant HTT (mHTT)-associated developmental impairments in neurogenesis may contribute to regional and cellular vulnerabilities to late selective neurodegeneration (Nguyen et al. 2013a; Nguyen et al. 2013b; Molero et al. 2016). Accordingly, transcriptomic studies have indicated that genes with key developmental and neural functions are preferentially impacted in the brain samples carrying mutant *HTT* (Jin et al. 2012; Achour et al. 2015; Labadorf et al. 2015).

In line with these findings, we have recently reported significant brain region-selective developmental abnormalities in seventy-five child and adolescent carriers of mutant *HTT* (van der Plas et al. 2019). Interestingly, cellular developmental identities have shown strong clinical correlates during the HD prodromal phase. For example, myelin breakdown and associated changes in ferritin distribution during the prodromal phase of disease closely mimics topographical profiles of cellular vulnerability to cell death occurring later in HD (Bartzokis et al. 2007). These findings support the notion that mutant *HTT-*associated developmental abnormalities are components of HD pathogenesis.

Mouse models are critical for advancing our understanding on disease mechanisms and developing novel treatments for HD (Leavitt et al. 2020; Brooks and Dunnett 2015; Chang et al. 2015; Farshim and Bates 2018; Kosior and Leavitt 2018). The important task of capturing neuropathological events in HD mouse models has been mostly carried out using imaging. While conventional histology allows sensitive detection of neuropathology at the cellular and molecular levels, it has limited coverage and cannot follow disease progression longitudinally. In comparison, magnetic resonance imaging (MRI) is a non-invasive imaging technique and provides a rich set of tissue contrasts and allows longitudinal tracking disease progression at the system level.

Here, we carried out for the first time a 3D morphometry analysis using our established segmentation tool that automatically reconstructs 30 different brain regions during postnatal development period in the heterozygous zQ175 HD mouse model, this model is widely used and displays adult-onset progressive behavioral deficits and brain pathology (Smith et al. 2014). It is well-known that mice expressing mutant HTT with CAG length within the range of the human adult-onset HD do not display behavioral and pathological phenotypes, most HD mouse models expressing m*HTT* with larger CAG repeat to recapitulate the pathological changes in HD (William Yang and Gray 2011). The differences in the sensitivity to mHTT between mice and human may be attributable to the influences of protein context and/or a difference in regulatory sequences between the mouse Htt and human HTT genes (Ehrnhoefer et al. 2009). The rationale for modeling human disorders in a non-human organism is to identify fundamental pathogenic mechanisms. In this regard, the heterozygous zQ175 knock-in (KI) HD model expresses m*HTT* in the most appropriate genomic context, since it has a human expanded CAG sequence inserted into the endogenous mouse *Htt* exon-1 region. The heterozygous zQ175 KI mouse model displays motor deficits after 6 months of age (Smith et al. 2014). Our current findings provide the first evidence for existing altered brain developmental processes at the system level in the heterozygous zQ175 KI HD model. In line with our observation in the human HD study (van der Plas et al. 2019), these data indicate new aspects of the HD pathogenesis which is important to guide future therapeutic development.

## Materials and Methods

### Mice

Twelve heterozygous zQ175 mice (6 males and 6 females) and 12 wild-type littermate controls (WT, 6 males and 6 females) were used for the longitudinal MRI scans, the breeding colony was purchased from Jackson Lab (Bar Harbor, ME) and in C57Bl/6 background strain. Genotyping and CAG repeat size were determined at Laragen Inc. (Culver City, CA, USA) by PCR of tail snips. The CAG repeat length was 223 ± 3 in male zQ175 mice and 225 ± 3 in female zQ175 mice used in the study. The heterozygous zQ175 KI HD mice have expected age of onset of motor deficits and brain atrophy after 6 months of age, these mice do not have significantly altered lifespan or exhibit seizures. All mice were housed at 3–5 mice per cage under specific pathogen-free conditions with a reversed 12-h light/dark cycle maintained at 23°C and provided with food and water ad libitum. All longitudinal scans were conducted in the mouse’s dark phase. This study was carried out in strict accordance with the recommendations in the Guide for the Care and Use of Laboratory Animals of the National Institutes of Health and approved by Institutional Animal Care and Use Committee of the Johns Hopkins University. The protocol was approved by the Committee on the Ethics of Animal Care and Use Committee (Permit Number: MO18M192). All procedures were performed under isoflurane anesthesia, and all efforts were made to minimize suffering. All mice are survived through longitudinal MRI scans.

### In Vivo Structural MRI Acquisition

*In vivo* MRI was performed on a vertical 9.4 Tesla MR scanner (Bruker Biospin, Billerica, MA, USA) with a triple-axis gradient and a physiological monitoring system (EKG, respiration, and body temperature). Mice were anesthetized with isoflurane (1%) mixed with oxygen and air at 1: 3 ratios via a vaporizer and a facial mask and scanned longitudinally (the same mice were imaged repeatedly over a 12-month period). We used a 20-mm diameter volume coil as the radiofrequency transmitter and receiver. Temperature was maintained by a heating block built into the gradient system. Respiration was monitored throughout the entire scan.

High-resolution anatomical images were acquired by using a three-dimensional (3D) T2-weighted fast spin echo sequence with the following parameters: echo time (TE)/repetition time (TR) = 40/700 ms, resolution = 0.1 mm × 0.1 mm × 0.25 mm, echo train length = 4, number of average = 2, and flip angle = 40°. Multi-slice T2-weighted images of the mouse brain were acquired by the RARE (Rapid Acquisition with Refocused Echoes) sequence with the following parameter (echo time (TE)/repetition time (TR) = 40 ms/1500 ms, RARE factor = 8, in-plane resolution = 0.125 mm x 0.125 mm, slice thickness = 1 mm, total imaging time less than 2 min) and used for high resolution anatomical imaging. Total imaging time was about 50 min per mouse. Mice recovered quickly once the anesthesia was turned off, and all mice survived the imaging sessions.

### Structural MRI Image Analysis

Images were first rigidly aligned to a template image by using automated image registration software (http://bishopw.loni.ucla.edu/AIR5/, AIR). The template image was selected from one of the images acquired from age-matched littermate control mice (mouse had the medium brain volume among the control group), which had been manually adjusted to the orientation defined by the Paxinos atlas with an isotropic resolution of 0.1 mm x 0.1 mm x 0.1 mm per pixel. After rigid alignment, images had the same position and orientation as the template image, and image resolution was also adjusted to an isotropic resolution of 0.1 mm × 0.1 mm × 0.1 mm per pixel. Signals from non-brain tissue were removed manually (skull-stripping). Skull-stripped, rigidly aligned images were analyzed by using Landmarker software (www.mristudio.org). Intensity values of the gray matter, white matter, and cerebral spinal fluid were normalized to the values in the template images by using a piece-wise linear function. This procedure ensured that subject image and template image have similar intensity histograms. The intensity-normalized images were submitted by Landmarker software to a linux cluster, which runs Large Deformation Diffeomorphic Metric Mapping (LDDMM). The transformations were then used for quantitative measurement of changes in local tissue volume among different mouse brains, by computing the Jacobian values of the transformations generated by LDDMM. There are about 30 different brain regions segmented automatically.

### Western Blotting

Brain tissue samples were homogenized in a buffer containing 50 mM Tris–HCl, pH 8.0, 150 mM NaCl, 0.1% (w/v) SDS, 1.0% NP-40, 0.5% sodium deoxycholate and 1% (v/v) protease inhibitor mixture. For SDS–PAGE, 10–20 μg of proteins were separated in a 4–20% gradient gel and transferred to a nitrocellulose membrane. The membrane was blotted with the following primary antibodies: anti-MBP (1:500), anti-MOG (1:1000), anti-C1QC (1:1000), and mouse anti-β-actin (Sigma, 1:5000). After incubation with HRP-conjugated secondary antibodies, the bound antibodies were visualized by chemiluminescence.

### Statistical Analysis

The brain segmentation volumes in male and female mice were analyzed separately. In order to estimate the trajectory of brain volume change with age, we employed quadratic polynomial models which will impose inflection points. The peaks can be interpreted as the characteristic points of mouse brain development. Two-way repeated measures (age and genotype) ANOVA were performed to examine the statistical difference between two groups along with age growth. Student’s *t*-test was used to measure the significant levels between WT and zQ175 HD groups at each givenage.

### Data Availability

The raw data supporting the findings reported here can be made available upon reasonable request.

## Results

### The Age Selection and CAG Size in zQ175 Mice

zQ175 heterozygous HD mice and their littermate controls at 3 ages, 3-, 5-, and 7- weeks old, were selected for the longitudinal MRI scans. These 3 ages represent the peak of myelination, synaptic pruning, and early adult stage respectively and are approximately equal to human 3-, 11-, and 18 years old (Semple et al. 2013). In order to avoid CAG expansions with passages, we rotated female HD and male HD mice for breeding. The CAG size in our original mouse colony obtained from the Jackson Laboratory is about 220. Our breeding strategy prevented the further CAG expansions, the CAG length ranges within 3 CAG size difference in all experimental HD mice used in this study (220 ± 3).

### zQ175 Mice Recapitulate the Region-Specific Developmental Abnormality of Human HD Brain in a Gender-Dependent Manner

**Figure 1:**
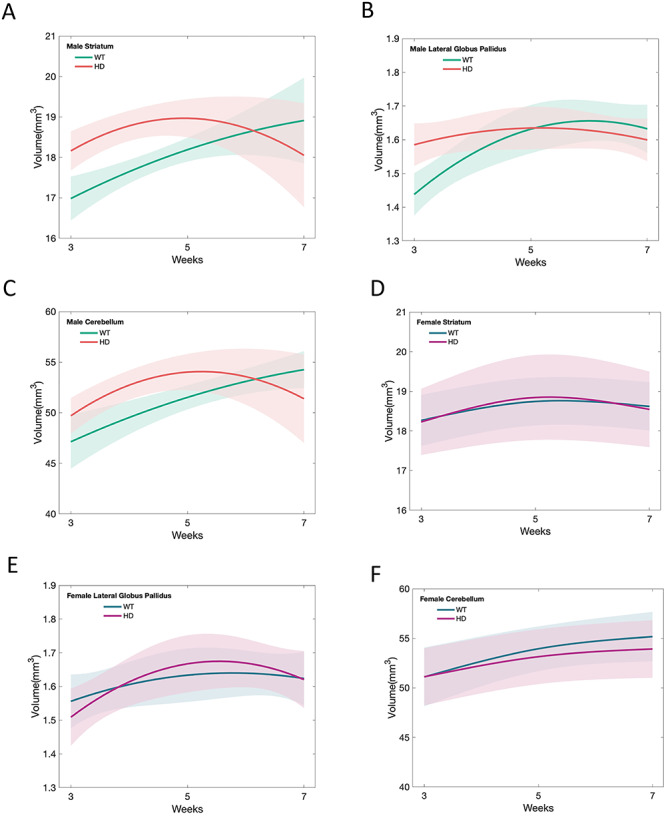
Longitudinal fitting curves of brain volume development in male zQ175 HD mice and their littermate controls. (**A**) Mean estimated age-dependent change of striatal volume in the male zQ175 mice (red) and control (green) groups. (**B**) Mean estimated age dependent change of the globus pallidus volume during postnatal development period in the male zQ175 mice (red) and control (green) groups. (**C**) Mean estimated age dependent change of the cerebellum volume during postnatal development period in the male zQ175 mice (red) and control (green) groups. Volume difference (y-axis) between HD mice and controls across age (x-axis), along with 95% confidence limits of the difference scores. (**D**) Mean estimated age-dependent change of striatal volume in the female zQ175 mice (pink) and control (blue) groups. (**E**) Mean estimated age dependent change of the globus pallidus volume during postnatal development period in the female zQ175 mice (pink) and control (blue) groups. (**F**) Mean estimated age dependent change of the cerebellum volume during postnatal development period in the female zQ175 mice (pink) and control (blue) groups.

Over the postnatal developmental period, there was a significant, non-linear difference between male zQ175 HD group and age-matched control group in age trajectories of the striatum. The male wild type (WT) mice exhibited gradual growth in the striatum from 3 weeks to 7 weeks of age; this pattern of normal development has been reported previously (Semple et al. 2013). In contrast, the developmental trajectory for the zQ175 male mice was strikingly different. These HD mice exhibited significant striatal hypertrophy prior to 5 weeks of age, compared to their littermate WT group (Fig. 1*A*, Tables 1 and 2). Then, between age 5 and 7 weeks, the striatal volume steadily decreased in the male zQ175 HD mice, but continued to increase in the WT group (Fig. 1*A*, Tables 2 and 3). The developmental abnormalities in the striatal volume of male zQ175 mice (Fig. 1, Table 4) recapitulated the age trajectories in human HD caudate-putamen development (van der Plas et al. 2019).

**Table 1:** Regional brain volumes in 3-week-old male zQ175 HD mice and their littermate controls

	WT			zQ175 HD			
	Mean	SD	95% CI	Mean	SD	95% CI	p-Value
LGP	1.438	0.071	(1.364, 1.512)	1.585	0.085	(1.496, 1.674)	0.009^*^
Striatum	16.984	0.541	(16.416, 17.552)	18.162	0.482	(17.656, 18.668)	0.003^*^
Cerebellum	47.136	2.653	(44.351, 49.92)	49.703	1.762	(47.854, 51.552)	0.081
Thalamus	20.495	0.786	(19.671, 21.32)	21.317	0.708	(20.575, 22.06)	0.086
Neocortex	85.179	5.06	(79.869, 90.489)	88.272	3.349	(84.757, 91.786)	0.245
Accumbens Nu	1.522	0.088	(1.43, 1.614)	1.62	0.058	(1.559, 1.681)	0.050

Note: ^*^P < 0.05 compared to the value of WT group by Standard Student’s t-test.

**Table 2:** Regional brain volumes in 5-week-old male zQ175 HD mice and their littermate controls

	WT			zQ175 HD			
	Mean	SD	95% CI	Mean	SD	95% CI	p-Value
LGP	1.632	0.07	(1.558, 1.705)	1.635	0.032	(1.601, 1.668)	0.918
Striatum	18.19	0.293	(17.882, 18.498)	18.968	0.471	(18.473, 19.462)	0.008^*^
Cerebellum	51.52	1.193	(50.268, 52.772)	54.021	1.791	(52.141, 55.901)	0.020^*^
Thalamus	20.764	0.613	(20.12, 21.408)	21.55	0.759	(20.754, 22.346)	0.078
Neocortex	90.21	1.031	(89.128, 91.293)	92.74	0.997	(91.693, 93.787)	0.002^*^
Accumbens Nu	1.588	0.047	(1.539, 1.638)	1.71	0.104	(1.6, 1.819)	0.036^*^

Note: ^*^P < 0.05 compared to the value of WT group by Standard Student’s t-test.

Like the striatum, a significant age-dependent brain regional volume difference during the postnatal brain developmental period between male zQ175 and their littermate controls was observed in the lateral globus pallidus (LGP), which is another vulnerable brain region in HD. The male WT mice exhibited gradual growth and reached to a plateau after 5 weeks of age (Fig. 1*B*, Tables 1 and 2). zQ175 male mice displayed LGP hypertrophy at 3 weeks of age, did not show the normal growth curve, then the volume of LGP slightly and gradually declined after 5 weeks of age (Fig. 1*B*, Tables 2 and 3). These abnormal developmental patterns in the LGP of zQ175 male mice (Table 4) are similar to those we observed in human HD brain MRI study (van der Plas et al. 2019).

**Table 3:** Regional brain volumes in 7-week-old male zQ175 HD mice and their littermate controls

	WT			zQ175 HD			
	Mean	SD	95% CI	Mean	SD	95% CI	p-Value
LGP	1.633	0.095	(1.533, 1.732)	1.6	0.087	(1.508, 1.691)	0.544
Striatum	18.911	1.059	(17.8, 20.021)	18.051	1.283	(16.705, 19.397)	0.235
Cerebellum	54.261	1.83	(52.34, 56.182)	51.385	4.37	(46.799, 55.971)	0.182
Thalamus	21.489	1.306	(20.119, 22.86)	20.325	1.404	(18.852, 21.799)	0.168
Neocortex	90.373	2.572	(87.674, 93.072)	88.927	3.559	(85.191, 92.662)	0.44
Accumbens Nu	1.617	0.1	(1.511, 1.722)	1.587	0.128	(1.453, 1.721)	0.666

**Table 4:** Statistical results in male zQ175 mice and their littermate controls

	Analysis of Variance	Two-way repeated measures ANOVA				
	***Source of Variation***	***SS***	***df***	***MS***	***F***	***P-value***
Striatum	Age	7.3846	2	3.6923	6.2160	0.0080
	Group + Age	6.9916	2	3.4958	5.8852	0.0098^*^
	Residuals	11.8800	20	0.5940		
LGP	Age	0.1039	2	0.0520	13.3141	0.0002
	Group + Age	0.0545	2	0.0272	6.9784	0.0050^*^
	Residuals	0.0781	20	0.0039		
Cerebellum	Age	153.2964	2	76.6482	13.9630	0.0002
	Group + Age	58.5483	2	29.2742	5.3329	0.0139^*^
	Residuals	109.7878	20	5.4894		
Thalamus	Age	0.5008	2	0.2504	0.2239	0.8014
	Group + Age	7.7467	2	3.8733	3.4625	0.0511
	Residuals	22.3733	20	1.1187		
Neocortex	Age	137.7731	2	68.8865	8.3401	0.0023
	Group + Age	36.7247	2	18.3623	2.2231	0.1343
	Residuals	165.1938	20	8.2597		
Accumbens Nu	Age	0.0371	2	0.0185	1.8578	0.1820
	Group + Age	0.0394	2	0.0197	1.9748	0.1649
	Residuals	0.1996	20	0.0100		

Note: ^*^P < 0.05 compared to the value of WT group by Standard Student’s t-test.

Interestingly, the cerebellum development in the male zQ175 mice also exhibited the similar abnormalities, with initial hypertrophy before 5 weeks of age, compared to their littermate WT group (Fig. 1*C*, Tables 1 and 2), then showed sharp decline by 7 weeks of age while WT mice demonstrated continuous increased volume (Fig. 1C, Tables 2 and 3). The findings are different from human HD cerebellum development pattern from our previous study.

In other 3 brain regions, thalamus, neocortex and nucleus accumbens, we observed similar developmental patterns, with initial increased volume and a later rapid decline in the volume in male zQ175 mice comparing to age-matched controls (Tables 1–4). However, the overall p values of statistical difference did not reach significance. In addition, there is no significant difference in the brain volume development between the female zQ175 mice and their litter mate controls (Fig. 1*D-F* and Supplementary Tables 1–4).

### Altered Myelin Development was Evident in Male zQ175 HD Mouse Brain

**Figure 2:**
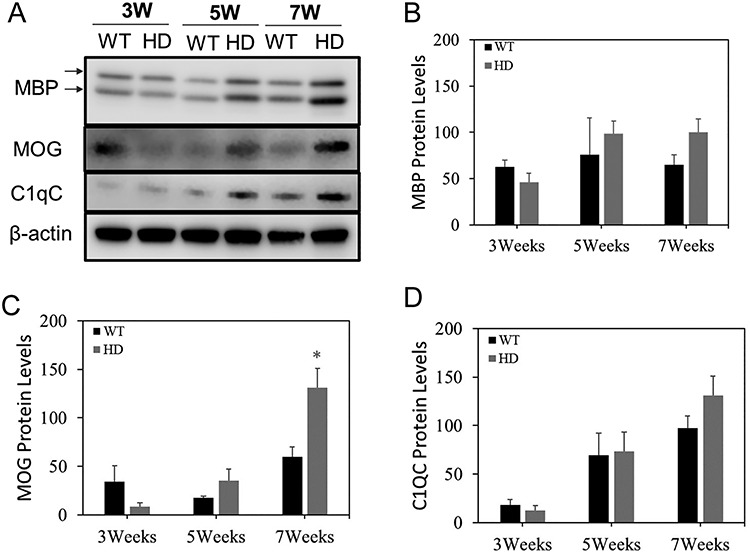
Abnormal myelin development in the heterozygous zQ175 knock-in mice. (**A**) Representative Western blots of MBP (2 different isoforms), MOG, and C1QC in the striatum of zQ175 (HD) mice and age-matched wild-type littermate (WT) controls at postnatal 3-, 5-, and 7- weeks of age. (**B-D**) Quantification of densitometry of indicated proteins in the Western blots. n = 3. *p < 0.05 compared with the values of WT mice by Student’s t-tests.

To determine the molecular basis of abnormal striatal volume development in male zQ175 mice, we examined the markers of myelin development, including the levels of myelin basic protein (MBP) and myelin oligodendrocyte glycoprotein (MOG). Both myelin protein levels were lower in the striatum of zQ175 mice at 3 weeks of age comparing to their age-matched control mice (Fig. 2*A-C*), while the levels of these myelin proteins had trend to increase with a statistical significance in the MOG levels at 7 weeks old zQ175 mice (Fig. 2*C*). C1QC is highly expressed in microglia and is a marker of synaptic pruning. We did not find the difference in the levels of C1QC between zQ175 mice and their age-matched controls (Fig. 2*D*). These results suggest that the difference in the postnatal striatal volume development between zQ175 HD mice and control mice may be due to altered myelination rather than changes in the synaptic pruning.

## Discussion

This is the first longitudinal MRI study of HD mouse brain development at 3D neuroimaging morphology. The motivation for this study was two-fold. First, it was prompted by a series of cellular and molecular findings that has established brain developmental abnormalities contribute to HD pathogenesis (Arteaga-Bracho et al. 2016; Nguyen et al. 2013a; Nguyen et al. 2013b; Molero et al. 2016; Mehler et al. 2019). Second, it was motivated by our recent neuroimaging study in pre-HD child and adolescent carriers of *mHTT* (van der Plas et al. 2019), in which we demonstrated that developmental trajectories in the caudate putamen and globus pallidus were markedly different between the *mHTT* carrier and control group (van der Plas et al. 2019). Remarkably, the current longitudinal analysis revealed a substantial difference in the brain volume growth of specific brain regions, namely striatum, globus pallidus, and cerebellum, in male zQ175 mice comparing to their littermate controls.

Genes associated with neurodegenerative diseases are normally expressed throughout neural development and are essential for the maintenance of neuronal subpopulations. *HTT* mutation may compromise defined subsets of these neural specification events in subtle ways that initially lead to impairments in the cellular homeostasis of evolving regional neuronal subpopulations, and adult-onset neuronal death. The observations of altered brain volume development in the present study, combining with the previous findings from cellular and molecular studies (Mehler and Gokhan 2000; Godin et al. 2010; Nguyen et al. 2013b; Molero et al. 2016) as well as our MRI study of human HD (van der Plas et al. 2019), support a developmental component in HD which may make neurons more vulnerable and selectively degenerated in the later life. The findings from both preHD children and zQ175 KI HD mouse model indicate that larger than normal volumes of selective brain regions are present at early postnatal HD brain development. We also observed an alter myelination in the male zQ175 mouse brain. Moreover, ‘precocious’ development leading to larger than normal volumes is commonly seen as developmental aberration (Shriver et al. 2006; Amaral et al. 2017), supporting the notion that these regions are structurally and functionally vulnerable which makes them prone to early degeneration. Longitudinal follow-up study of a larger cohort of mice with neuroimaging and molecular profiling may help to elucidate novel mechanisms underlying the development contribution to HD pathogenesis.

Our results illuminate a postnatal developmental window associated with HD pathogenesis that may have major implications for our understanding of the process governing the neurodegeneration in HD. These observations have the potential to identify new classes of biomarkers and innovative therapeutic targets to delay, reverse, or prevent the onset and progression of HD. In fact, HD has a long interval before clinical manifestation during which therapeutic strategies may be introduced and provide the most effective outcome for patients. Our current findings in a faithful mouse model and the previous study in preHD child and adolescent mutant HTT carriers (van der Plas et al. 2019) suggest that brain region-specific profiles of degeneration are likely due to regional cellular vulnerabilities programmed during early development.

The neuropathological process may have started during a period of development preceding cell death in HD. Determining when such decline begins and the selective vulnerability of specific brain region(s) to the degenerative cascade is important for development of disease-modifying therapies. In addition, mHTT not only alters the function of its protein partners, but may also turn mHTT into a dominant-negative factor, further disrupting the developmental functions of the wild type HTT allele (Feero and Hoffman 1995; Rubinsztein 2003; Rubinsztein and Carmichael 2003). HTT is a pleiotropic protein with functional roles in regulating neuronal survival, transcription, and metabolism (Cattaneo et al. 2005), mHTT may deregulate the entire process of stem cell-mediated neurogenesis and gliogenesis through impairments in the transcription (Feero and Hoffman 1995), such that mHTT can directly affect metabolic profiles in premanifest HD by altering mitochondrial biogenesis, maintenance, and function at the transcriptional level by inhibiting Peroxisome proliferator-activated receptor gamma coactivator 1-alpha (PGC1α) (Cui et al. 2006).

The principal HD neuropathology is classically conceptualized as degeneration of striatal medium spiny neurons (MSNs) (Ross and Tabrizi 2011; McColgan and Tabrizi 2018). In addition, globus pallidus is a primary projection target of the striatum and dramatically affected and degenerated in HD (Ross and Tabrizi 2011; Singh-Bains et al. 2016). Our results indicate that mHTT may underly the observed similarities between striatal and globus pallidus developmental trajectories in HD mice. These results support that neurodegenerative diseases could begin with aberrant brain development in regional neuronal subpopulations (Mehler and Gokhan 2000). In fact, molecular studies in mice have demonstrated that degeneration of MSNs is preceded by abnormal development of these cells (Mehler and Gokhan 2000; Molero et al. 2016; Wiatr et al. 2018). The observed difference in the brain volume trajectory of striatal and globus pallidus in male zQ175 HD mice and altered myelination during postnatal development period further strengthens the notion of abnormal neural development inHD.

We also noticed altered volume development in the cerebellum of male zQ175 mice, similar to the other 2 most affected brain regions, striatum and globus pallidus, with initial hypertrophy and rapid volume decline, while our recent human HD brain developmental study indicates that volume of the cerebellum did not follow this pattern (van der Plas et al. 2019). Interestingly, in that same subjects, functional connectivity analysis showed that the cerebellum was hyper-connected to the striatum, suggesting that mHTT directs cerebellar-striatal circuitry formation (Tereshchenko et al. 2020). This finding also supports that the cerebellum may play a compensatory role. In our previous study of Juvenile-onset HD, the cerebellum in the adult age was proportionally enlarged while the absolute value of cerebellum volume did not show significant difference, this pattern was also consistent across 4 mouse models of HD, including zQ175 model (Tereshchenko et al. 2019). That study included both male and female mice. Although the current analysis suggests that the cerebellum begins to decrease in volume in early life (males only), the analysis at adult age (6 months) in our previous study (Tereshchenko et al. 2019) suggests that this volumetric trajectory does not continue as in other 2 brain regions, and in fact stabilizes with the end result being proportional enlargement. Cerebellum is notably “spared” in comparison with other brain regions in adult age, but evidence has suggested that cerebellar abnormalities are present in HD (Rosas et al. 2003). Until now, inconsistent reports of the cerebellum volume in HD have emerged from the literature; autopsy data suggest that atrophy is most marked within the cortical gray matter and gray matter of the deep cerebellar nuclei (Rodda 1981; Jeste et al. 1984; Fennema-Notestine et al. 2004; Rub et al. 2013; Ruocco et al. 2006), whereas structural imaging data (voxel-based morphometry) localize the most significant volume changes to the cerebellar white matter or detect no disease effects, one study in pre-manifest HD has detected significant cerebellar volume loss whereas others have not (Gomez-Anson et al. 2009; Hobbs et al. 2010). A more focused, multimodal imaging analysis of the cerebellum in a larger cohort and longitudinal study covering from the early development stage to the symptomatic stage is warranted.

It is worth to mention that we observed a gender-dependent difference in the zQ175 HD mouse brain volume development, a finding that was not present in the human preHD childhood sample. Male zQ175 HD mice recapitulated the abnormal development patterns in the striatum and globus pallidus observed in human HD, but female HD mice did not show the significant difference in the brain development in all regions we tested (Fig. 1*D-F* and Supplementary Tables 1–4). The mechanisms underlying this gender-dependent differences require further investigation. A recent study reported a large proportion of mammalian traits both in wildtype and mutants are influenced by sex (Karp et al. 2017). This result has implications for interpreting disease phenotypes in animal models. Interestingly, sex-specific effects of HTT on brain development for CAG repeat ranges below disease threshold has been reported (Lee et al. 2017). Normal variation in CAG repeat length was associated with different putamen and cerebellum volume in male participants. Gender-dependent difference was also reported in a rat HD model in glucose uptake across various brain regions (Reilmann et al. 2015). Our previous study in the homozygous zQ715 found gender-dependent differences in motor performance (Peng et al. 2016). These results suggest that sex should be considered in the experimental design and data analysis when we use this model. Our findings further emphasize that data from male and female zQ175 mice need to be analyzed separately and sex as a biological variable should be considered in experimental design and result interpretation.

Our results highlight a critical need for research in understanding the brain developmental effects and mechanism in HD. The knowledge obtained in this aspect will be important for optimization of disease modifying therapy and prevention. Strategies to prevent disease onset may have to consider the brain developmental contribution and introduce treatment to individuals at a young age. Modulation of pathways involved in brain development may represent a new therapeutic direction. The potential effect of gene therapies targeting on neurodevelopmental event is worth to consider for HD therapeutic intervention.

## Notes

We are thankful for support from the National Institute of Health. *Conflict of Interest:* Dr Susumu Mori co-owns ``AnatomyWorks''. Susumu Mori is its CEO. This arrangement is being managed by the Johns Hopkins University in accordance with its conflict-ofinterest policies. All other authors declare no conflict of interests.

## Funding

Contract fund from the Department of Psychiatry, University of Iowa Carver College of Medicine. National Institute of Health R01NS082338 (to W.D), R21NS104480 (to J.H. and W.D), and China Scholarship Council scholarship (to Q.W).

## Supplementary Material

Zhang_et_al_MS-CCC-2020-00046R1-Supplemental_Data_tgaa044
