# Supplementary material for: Abnormal Brain Development in Huntington’ Disease Is Recapitulated in the zQ175 Knock-In Mouse Model
Source: Cereb Cortex Commun. 2020 Aug 5;1(1):tgaa044. doi: 10.1093/texcom/tgaa044 (PMC7501464; doi:10.1093/texcom/tgaa044)
Supplement: Zhang_et_al_MS-CCC-2020-00046R1-Supplemental_Data_tgaa044 [file zhang_et_al_ms-ccc-2020-00046r1-supplemental_data_tgaa044.docx]

**Abnormal brain development in Huntington’ disease is recapitulated in the zQ175 knock-in mouse model**

Chuangchuang Zhang^1^, Qian Wu^1,2^, Hongshuai Liu^1^, Liam Cheng^1^, Zhipeng Hou^3^, Susumu Mori^3^, Jun Hua ^3,4^, Christopher A. Ross ^1,5,6,7^, Jiangyang Zhang^8^, Peggy C. Nopoulos^9^, Wenzhen Duan^1,5,10^*

^1^Division of Neurobiology, Department of Psychiatry and Behavioral Sciences, Johns Hopkins University School of Medicine, Baltimore, MD, USA. ^2^Beijing University of Chinese Medicine, Beijing, China. ^3^Department of Radiology, Johns Hopkins University School of Medicine, Baltimore, MD, USA. ^4^F.M. Kirby Research Center for Functional Brain Imaging, Kennedy Krieger Institute, Baltimore, MD, USA. ^5^Department of Neuroscience, Johns Hopkins University School of Medicine, Baltimore, MD, USA. ^6^Department of Pharmacology and Molecular Sciences, Johns Hopkins University School of Medicine, Baltimore, MD, USA. ^7^Department of Neurology, Johns Hopkins University School of Medicine, Baltimore, MD, USA. ^8^Deaprtment of Radiology, New York University Grossman School of Medicine, ^9^Departments of Psychiatry, Neurology, Pediatrics, University of Iowa Carver College of Medicine, Iowa city, IA, USA.  ^10^Program in Cellular and Molecular Medicine, Johns Hopkins University School of Medicine, Baltimore, MD, USA.

* **Correspondence to**: Wenzhen Duan, Division of Neurobiology, Department of Psychiatry and Behavioral Sciences, Johns Hopkins University School of Medicine. CMSC 8-121, 600 North Wolfe Street, Baltimore, MD 21287.

Tel: 410-502-2866; Fax: 410- 614-0013. Email: [wduan2@jhmi.edu](mailto:wduan2@jhmi.edu).

**Supplemental Table 1.** Regional brain volumes in 3-week-old female zQ175 HD mice and their littermate controls.

|  | **WT** | | | **zQ175 HD** | | | |
| --- | --- | --- | --- | --- | --- | --- | --- |
|  | Mean | SD | 95% CI | Mean | SD | 95% CI | p-Value |
| **Brain** | 427.53 | 11.5 | (415.465, 439.6) | 424.436 | 13.098 | (410.69, 438.181) | 0.673 |
| **striatum** | 18.167 | 0.652 | (17.483, 18.851) | 18.076 | 0.774 | (17.264, 18.888) | 0.831 |
| **Neocortex** | 89.873 | 7.307 | (82.205, 97.541) | 89.206 | 5.419 | (83.519, 94.893) | 0.861 |
| **Cerebellum** | 50.837 | 3.121 | (47.561, 54.112) | 50.913 | 2.924 | (47.844, 53.982) | 0.966 |
| **Thalamus** | 21.827 | 1.668 | (20.076, 23.577) | 21.214 | 1.584 | (19.552, 22.876) | 0.529 |
| **LGP** | 1.622 | 0.182 | (1.431, 1.814) | 1.513 | 0.087 | (1.422, 1.604) | 0.225 |
| **Accumbens Nu** | 1.63 | 0.108 | (1.517, 1.743) | 1.597 | 0.075 | (1.518, 1.675) | 0.547 |

**Supplemental Table 2**. Regional brain volumes in 5-week-old female zQ175 HD mice and their littermate controls.

|  | **WT** | | | **zQ175 HD** | | | |
| --- | --- | --- | --- | --- | --- | --- | --- |
|  | Mean | SD | 95% CI | Mean | SD | 95% CI | p-Value |
| **Brain** | 457.394 | 15.411 | (441.222, 473.567) | 449.585 | 18.024 | (430.67, 468.5) | 0.439 |
| **striatum** | 18.945 | 0.59 | (18.326, 19.565) | 19.152 | 1.191 | (17.902, 20.403) | 0.714 |
| **Neocortex** | 92.925 | 3.764 | (88.975, 96.875) | 92.925 | 4.455 | (88.251, 97.6) | 1 |
| **Cerebellum** | 54.49 | 1.942 | (52.453, 56.528) | 53.57 | 2.682 | (50.756, 56.384) | 0.513 |
| **Thalamus** | 21.294 | 0.533 | (20.735, 21.853) | 21.891 | 1.581 | (20.232, 23.55) | 0.414 |
| **LGP** | 1.633 | 0.061 | (1.569, 1.698) | 1.665 | 0.095 | (1.565, 1.765) | 0.515 |
| **Accumbens Nu** | 1.635 | 0.078 | (1.569, 1.698) | 1.65 | 0.117 | (1.527, 1.772) | 0.811 |

**Supplemental Table 3**. Regional brain volumes in 7-week-old female zQ175 HD mice and their littermate controls.

|  | **WT** | | | **zQ175 HD** | | | |
| --- | --- | --- | --- | --- | --- | --- | --- |
|  | Mean | SD | 95% CI | Mean | SD | 95% CI | p-Value |
| **Brain** | 464.065 | 14.144 | (449.222, 478.908) | 460.929 | 10.62 | (449.784, 472.074) | 0.674 |
| **striatum** | 18.52 | 0.619 | (17.87, 19.17) | 18.392 | 0.894 | (17.454, 19.33) | 0.78 |
| **Neocortex** | 91.889 | 3.737 | (87.968, 95.811) | 90.316 | 4.242 | (85.864, 94.768) | 0.511 |
| **Cerebellum** | 54.903 | 2.659 | (52.113, 57.693) | 53.716 | 2.952 | (50.618, 56.813) | 0.481 |
| **Thalamus** | 20.821 | 0.783 | (19.999, 21.642) | 20.927 | 1.237 | (19.628, 22.225) | 0.864 |
| **LGP** | 1.624 | 0.028 | (1.595, 1.653) | 1.62 | 0.083 | (1.532, 1.707) | 0.904 |
| **Accumbens Nu** | 1.589 | 0.094 | (1.49, 1.688) | 1.554 | 0.08 | (1.471, 1.638) | 0.503 |

**Supplemental Table 4. Statistical results in female zQ175 mice and their littermate controls.**

|  | ***Source of Variation*** | ***SS*** | ***df*** | ***MS*** | ***F*** | ***p-value*** |
| --- | --- | --- | --- | --- | --- | --- |
| **LGP** | Age | 0.0412 | 2 | 0.0206 | 1.8205 | 0.1878 |
|  | Greoup+Age | 0.0321 | 2 | 0.0161 | 1.4192 | 0.2653 |
|  | Residuals | 0.2263 | 20 | 0.0113 |  |  |
| **Striatum** | Age | 5.2918 | 2 | 2.6459 | 5.6788 | 0.0111 |
|  | Grepup+Age | 0.2022 | 2 | 0.1011 | 0.2170 | 0.8068 |
|  | Residuals | 9.3185 | 20 | 0.4659 |  |  |
| **Neocortex** | Age | 68.9175 | 2 | 34.4588 | 2.7784 | 0.0861 |
|  | Greoup+Age | 3.7415 | 2 | 1.8708 | 0.1508 | 0.8610 |
|  | Residuals | 248.0514 | 20 | 12.4026 |  |  |
| **Cerebellum** | Age | 87.3087 | 2 | 43.6544 | 11.2876 | 0.0005 |
|  | Group+Age | 2.6612 | 2 | 1.3306 | 0.3441 | 0.7130 |
|  | Residuals | 77.3495 | 20 | 3.8675 |  |  |
| **Thalamus** | Age | 3.7603 | 2 | 1.8801 | 1.6972 | 0.2085 |
|  | Group+Age | 2.2195 | 2 | 1.1097 | 1.0017 | 0.3849 |
|  | Residuals | 22.1564 | 20 | 1.1078 |  |  |
| **Accumbens Nu** | Age | 0.0305 | 2 | 0.0152 | 2.3733 | 0.1189 |
|  | Group+Age | 0.0047 | 2 | 0.0023 | 0.3652 | 0.6986 |
|  | Residuals | 0.1285 | 20 | 0.0064 |  |  |
